# Whole genome resequencing of the human parasite *Schistosoma mansoni* reveals population history and effects of selection

**DOI:** 10.1038/srep20954

**Published:** 2016-02-16

**Authors:** Thomas Crellen, Fiona Allan, Sophia David, Caroline Durrant, Thomas Huckvale, Nancy Holroyd, Aidan M. Emery, David Rollinson, David M. Aanensen, Matthew Berriman, Joanne P. Webster, James A. Cotton

**Affiliations:** 1 Department of Infectious Disease Epidemiology, Imperial College London, St Mary’s Campus, Norfolk Place, London W2 1PG, United Kingdom; 2 Wellcome Trust Sanger Institute, Hinxton, CB10 1SA, United Kingdom; 3 Department of Life Sciences, Natural History Museum, London, SW7 5BD, United Kingdom; 4 Department of Pathology and Pathogen Biology, Royal Veterinary College, University of London, Hertfordshire, AL9 7TA, United Kingdom

## Abstract

*Schistosoma mansoni* is a parasitic fluke that infects millions of people in the developing world. This study presents the first application of population genomics to *S. mansoni* based on high-coverage resequencing data from 10 global isolates and an isolate of the closely-related *Schistosoma rodhaini*, which infects rodents. Using population genetic tests, we document genes under directional and balancing selection in *S. mansoni* that may facilitate adaptation to the human host. Coalescence modeling reveals the speciation of *S. mansoni* and *S. rodhaini* as 107.5–147.6KYA, a period which overlaps with the earliest archaeological evidence for fishing in Africa. Our results indicate that *S. mansoni* originated in East Africa and experienced a decline in effective population size 20–90KYA, before dispersing across the continent during the Holocene. In addition, we find strong evidence that *S. mansoni* migrated to the New World with the 16–19^th^ Century Atlantic Slave Trade.

*Schistosoma mansoni* is a dioecious trematode (fluke) and an aetiological agent of the neglected tropical disease schistosomiasis, which infects over 250 million people and causes over 11 thousand deaths annually^1^,^2^. The trematode has a wide geographic range; the majority of *S. mansoni* infections are found in sub-Saharan Africa and Madagascar, though transmission foci also exist in the Arabian Peninsula, South America and the Caribbean. Chronic pathology is caused when eggs laid by the adult worms, residing in the mesenteric veins, are swept by the bloodstream into internal organs causing an inflammatory response that may result in fibrosis and calcification of the liver and spleen^3^. *Schistosoma mansoni* has a complex lifecycle with an intermediate freshwater-snail host (*Biomphalaria spp.*), consequently its epidemiology is closely tied with water contact and prevalence of the disease is greatest in communities that live close to endemic freshwater lakes and rivers^4^.

Since the sequencing of the 380 megabase (Mb) *S. mansoni* reference genome^5^, genomic resources have contributed to comparative^6^ and functional studies^7^, including the discovery of the molecular basis for oxamniquine resistance^8^, but little is known about genome-wide variation between parasites in the field. While large populations of *S. mansoni* have been investigated^9^,^10^,^11^, all previous studies in this field have been restricted to analysing a small number of markers, typically using the mitochondrial *cox*1 gene, nuclear ITS region or <10 microsatellite loci. In this study we present the first population genomic study of *S. mansoni* based on whole genome re-sequencing data for nine isolates collected from field sites across Africa and the New World. African individuals were selected from distinct clades based on previous phylogenetic data^10^ to represent the diversity of extant *S. mansoni*. We also include the NMRI (Puerto Rican) laboratory strain that was used to build the reference genome^5^. In addition we have sequenced *Schistosoma rodhaini,* a rodent schistosome species that is firmly established as the closest outgroup to *S. mansoni*^12^,^13^.

Whole-genome resequencing of populations allows us to explore how patterns of genetic variation change across the genome and thus detect the effects of selection. This can be of particular importance in pathogens where genomic methods can identify targets of balancing selection that interact with host immunesystems, for instance genes involved in host cell invasion in *Plasmodium spp.*^14^,^15^,^16^. Testing for coding sequences under strong positive selection may identify genes involved in longer-term selection in a coevolving host-parasite system^17^. To shed light on the divergence between *S. mansoni* and *S. rodhaini*, we catalogue the extensive genome-wide variation between a global collection of isolates and analyse this variation to better understand the emergence of the human-infective *S. mansoni* and its subsequent demographic and evolutionary history. Using a set of population genetic approaches, we look at how signatures of natural selection vary between different coding regions of these genomes, aiming to identify genes that may be involved in host-pathogen interaction or may have facilitated the adaptation of *S. mansoni* to human hosts.

Understanding the evolutionary origins of pathogens such as *S. mansoni* and their subsequent demographic history has implications, not only for a better understanding of the parasite itself, but also of human evolutionary history as infectious diseases have consistently been shown as a major force driving selection in human populations^18^,^19^. Despite its importance, little is known about either the origin or subsequent history of *S. mansoni*. Previous work based on interspecies divergence at two short loci estimated that the split between *S. mansoni* and *S. rodhaini* occurred somewhere between 1.2 and 10 million years ago (MYA), around the time that the hominin clade split from other great apes and that the human infective species emerged in West Africa^20^. This has been contested by the view that East Africa is the most likely origin of *S. mansoni* due to the “conspicuous amount of diversity” observed in sequence data from this region^9^,^21^,^22^. A subsequent study using a mitochondrial molecular clock suggested that the most recent common ancestor of extant East African *S. mansoni* occurred 0.3–0.43 MYA and that the divergence between *S. mansoni* and *S. rodhaini* was around 2.8MYA^21^.

Two main hypotheses have been proposed to explain the divergence between African and South American strains. One suggestion is that African and South American *S. mansoni* diverged along with their intermediate snail hosts *Biomphalaria pfeiffeiri* and *B. glabrata*, the split between which is approximately dated to the separation of the continents 80–120 millions years ago^23^,^24^. A second proposal is that the divergence between these two *S. mansoni* populations is much more recent as African populations entered the New World through the Atlantic Slave Trade of 16^th^–19^th^ Centuries^9^. This hypothesis has been supported by studies using DNA barcoding and microsatellite techniques, which have observed less genetic diversity among West African and New World *S. mansoni* suggestive of a more recent introduction of the parasite into these areas^21^,^22^.

In contrast to data from a small set of loci, whole genome data offers a more powerful and unbiased approach to reconstruct the history of a species as recombination gradually breaks up the genome into blocks that have independent ancestry. Genome-wide data thus provides many independent estimates of the coalescent process of ancestry and far greater precision in estimates of population history and population genetic parameters such as effective population sizes (*N*_*e*_) and recombination rates^25^. We used two different coalescent models that both make simplifications in order to approximate the coalescence under recombination from whole-genome sequences^26^. Pairwise Sequentially Markovian Coalescent (PSMC) uses the pattern of heterozygosity along a single diploid genome to estimate *N*_*e*_ through time^27^ and Generalised Phylogenetic Coalescent Sampler (G-PhoCS) uses data from 1 kb non-recombining windows across the genome so that the history of population divisions and migration between populations can be modeled^28^. These complementary methods have already been shown to infer the complex demographic history of species such as humans and great apes using a small number of individual genomes^29^, though this is the first study to apply these methods to sequence data from a human-infective parasite.

## Results

### Genome-wide intra- and inter-species diversity

We sequenced the genomes of 10 *Schistosoma mansoni* and one *S. rodhaini* isolate to a median of 46x coverage. Sequence data were mapped against the *S. mansoni* reference genome (v5.2)^30^; coverage varied between 23x and 58x across samples (Table 1). The proportion of total reads mapped and of ‘proper pairs’ (reads mapped in the correct orientation and distance) did not vary significantly between *S. mansoni* isolates (from 75–97% for total mapping, 70–91% for proper pairs) with the exception of isolate Guadeloupe 1 where fewer reads map against the reference (58% total mapped and 54% for proper pairs) due to contamination in the sample, whereby 24.8% of sequenced reads mapped against *Mus musculus*. Reads from *S. rodhaini* mapped well against the *S. mansoni* reference suggesting that SNPs between species did not affect placement of reads, but the proportion of proper pairs was lower, presumably due to structural differences altering the orientation and distance between read pairs between the two species (84% of *S. rodhaini* reads map and 56% as proper pairs). These results confirm that the reference assembly is suitable for studying variation between all of our isolates. Additional evidence that the *S. mansoni* assembly captures most of the *S. rodhaini* gene complement comes from examining the depth of reads mapping to coding sequences: of 10,722 coding sequences in *S. mansoni*, 10,003 were covered by a minimum of 10 × across at least 75% of the coding sequence in the *S. rodhaini* alignment.

Numbers of SNPs for each sequence, based on the intersection of three SNP calling and filtering algorithms (see Table 1; methods), show a general geographical gradient, with the number of SNPs increasing with distance from Puerto Rico, which is the origin of the NMRI reference isolate (Supplementary Fig. S1). The total number of SNPs per isolate (including both homozygous and heterozygous sites) ranged from <1 × 10^6^ across the New World and Senegal sequences, to ~1.5 × 10^6^ SNPs in Cameroon and ~2 × 10^6^ SNPs in Uganda. The outgroup species *S. rodhaini* has ~7 × 10^6^ fixed differences when called against the reference, representing an average of about one variant every 50 basepairs. A notable exception to this geographical trend was the Coastal Kenya sample, which showed a reduced number of SNPs (~8 × 10^5^), more comparable to West African and New World strains than the more divergent Uganda sequences.

### Population structure of *S. mansoni*

**Figure 1:**
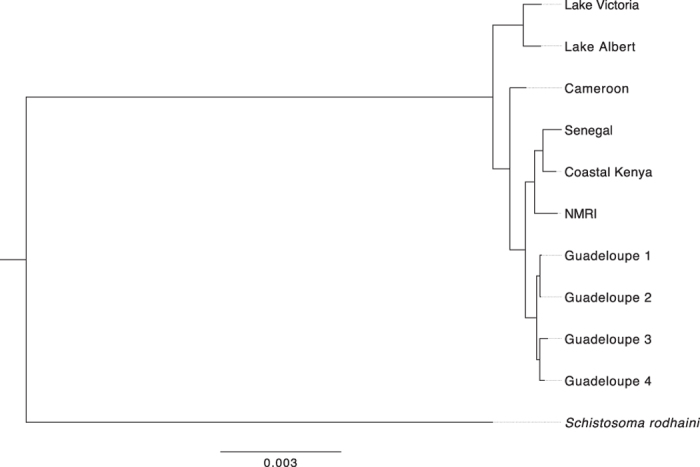
Maximum likelihood phylogeny of *Schistosoma* isolates based on whole-genome SNP data. Values on nodes are the number of bootstrap replicates supporting the split induced by that node*. S. rodhaini* has been set as the outgroup.

We used the identified SNPs across our eleven samples to understand the broad population genetic structure of *S. mansoni* and their relationship to *S. rodhaini*. A maximum likelihood phylogeny (Fig. 1) confirmed that *S. rodhaini* was highly divergent from the ten *S. mansoni* samples. Rooting the phylogeny with the *S. rodhaini* sample as an outgroup showed that the deepest split within our *S. mansoni* samples separated the Uganda isolates (Lake Victoria and Lake Albert, bootstrap support (BS) = 100) from our other samples, followed by the Cameroon samples (BS = 100). The rest of the sequences fell within a broader clade that included the Senegal, Coastal Kenya and all of the New World samples, suggesting a more recent divergence.

**Figure 2:**
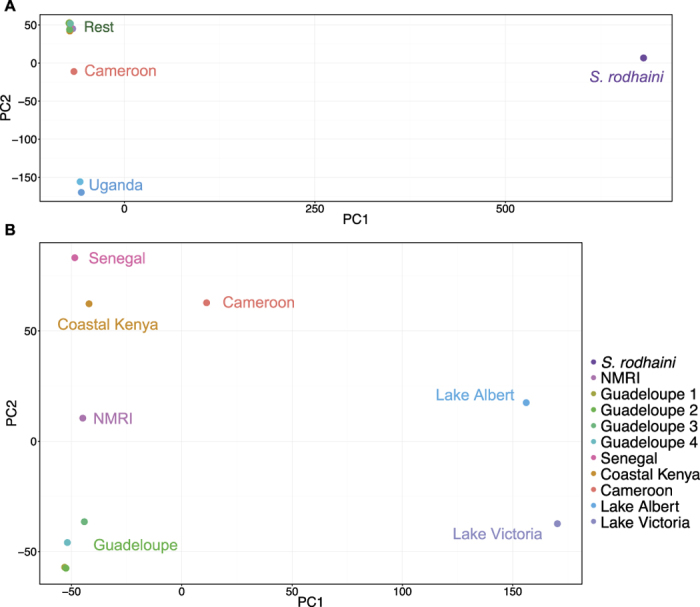
Principal component analyses of isolate SNP data. (**A**) including both *S. mansoni* and *S. rodhaini* samples (Variance represented by PC1: 72.5%, PC2: 9.9%) (**B**) *S. mansoni* samples only, showing more details of the relatedness of isolates within this species (Variance represented by PC1: 36.0%, PC2: 13.7%).

The phylogenetic pattern is supported by the principal component analysis (PCA) of SNPs (Fig. 2). Within the *S. mansoni* sequences, Uganda and, to a lesser extent, Cameroon samples are separated from the others by the first principal component (PC), which accounts for 36.0% of the variance. Isolates within the more recently formed clade are separated along the second principal component axis (13.7% of variance), which distinguishes the two African samples in this group (Senegal and Coastal Kenya) from the Guadeloupe and NMRI (Puerto Rico-derived) samples. As expected, incorporating *S. rodhaini* in a PCA separates this sample from all of the *S. mansoni* isolates by the first PC, which accounts for >70% of the variance and collapses the structure in the Senegal/Coastal Kenya/Guadeloupe group as a second PC only separates the Uganda and Cameroon *S. mansoni* from all other samples.

### Non-synonymous and synonymous changes

Tests for selection have identified interesting genes likely to be involved in host interaction in other parasites such as erythrocyte binding proteins in *P. falciparum* and *P. vivax*^15^,^31^, therefore our whole-genome data provides a unique opportunity to identify genes that are potentially host interacting in *S. mansoni*. We examined positive and negative selection between African *S. mansoni* and *S. rodhaini* by comparing *d*_*N*_*/d*_*S*_ using a branch model and balancing selection within *S. mansoni* using the Hudson-Kreitman-Aguade and McDonald-Kreitman tests (see below). The ratio of *d*_*N*_*/d*_*S*_ values across the filtered set of 9514 coding sequences was calculated with codeml using a branch model (see Methods), where we set the branch groups as African *S. mansoni* and *S. rodhaini* using the phylogenetic relationship in Fig. 1.

The branch *d*_*N*_*/d*_*S*_ values were centered around 1 (interquartile range: 0.44–1.1), examining the tails of the distribution we found 767 coding sequences showing evidence of positive selection (*d*_*N*_*/d*_*S*_ > 2) and 2167 genes under strong purifying selection (*d*_*N*_*/d*_*S*_ < 0.1). Detailed functional annotation is lacking for most genes in *S. mansoni*, nonetheless we explored whether we could detect any functionally enriched groups. Statistical overrepresentation analysis of the genes under positive selection showed an enrichment of gene ontology (GO) terms relating to the extracellular matrix (ECM) structural protein (GO:0005201, fold enrichment > 5, *p* = 2.42 × 10^−3^). These five genes (Smp_197370, Smp_159600, Smp_170340, Smp_196840, Smp_033250) each contain multiple pfam domains of collagen triple helix repeats. All five genes have corresponding RNA-seq evidence of a significant increase in expression during the 24-hour schistosomula stage of the life cycle in pairwise comparisons with both the 3-hour schistosomula and the adult worms^30^, implicating these genes in adaptation to the definitive human host. We formally tested whether *d*_*N*_*/d*_*S*_ values differed from 1, where coding sequences are assumed to be under drift rather than positive or negative selection (see Methods). Across all coding sequences, 52 were found to be significant (*p* < 0.05) of which 13 were under positive selection and 39 under purifying selection. Again, poor functional annotation limits to extent to which we could interpret these results, as 46 out of 52 significant coding sequences were unclassified. Despite this, we find evidence of significant positive selection on a number of genes involved in host-parasite interactions such as venom allergen-like (VAL) 21 gene (Smp_159290) and cercarial elastase gene (Smp_119130). Both of these genes are expressed in the cercarial (mammalian infective) stage of the lifecycle and are known to be host-interacting^32^,^33^. The full results are shown in Supplementary Table S1.

The *d*_*S*_ values on the *S. rodhaini* branch ranges from 0 to 0.82 with a median value of zero and only two coding sequences with tree length for *d*_*s*_ > 0.5, suggesting that saturation of synonymous mutations has not affected our results. Furthermore, methods based on modeling the codon substitution process have been shown to be more robust to saturation than alternative methods^34^,^35^.

### Hudson-Kreitman-Aguade & McDonald-Kreitman Tests

**Figure 3:**
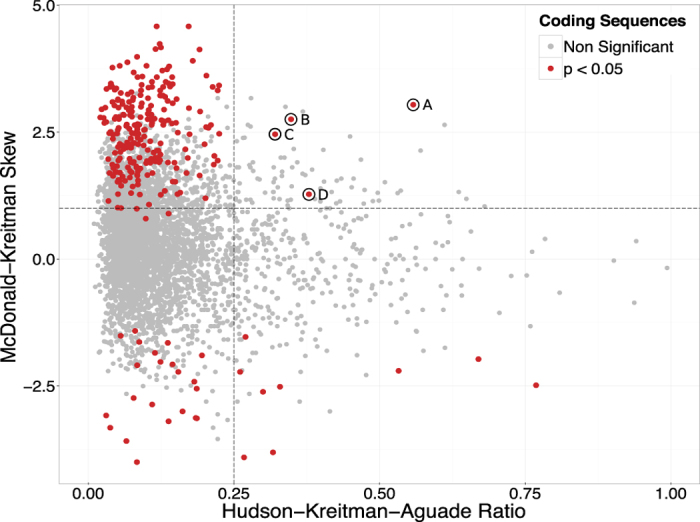
Population genetic evidence for balancing and directional selection on *S. mansoni* genes. McDonald-Kreitman Skew (MKS) and Hudson-Kreitman-Aguade ratio (HKAr) performed on 4147 coding sequences. Genes with a significant MKS (Fisher’s exact test, *p* < 0.05) are shown in red. We have selected a cutoff of HKAr > 0.25 and MKS > 1 to identify genes under balancing selection (hashed lines). Significant genes with a high HKAr and positive MKS are (**A**) Smp_035200- hsp40 protein (**B**) Smp_157450- uncharacterised protein (**C**) Smp_145220- Homeobox protein (**D**) Smp_160080- Elongator Complex Protein 6.

The Hudson-Kreitman-Aguade (HKA) test measures the ratio of polymorphisms (*π*) to fixed differences (*K*) across genes, whereby a higher HKA ratio (HKAr) reflects an enrichment of intra-specific variation that may be indicative of balancing selection or weak purifying selection. The mean value of *K* is 17.12 (pairwise nucleotide differences per kb) and the mean HKAr across genes is 0.132. The distribution of HKAr values is positively skewed with the majority of genes showing low values indicative of higher levels of fixed differences to polymorphisms (upper quartile = 0.15), as would be expected as the *S. rodhaini* outgroup is highly diverged. Therefore analysis of the few genes with high HKAr (>0.25) may be indicative of balancing selection (see Fig. 3 and below).

The McDonald-Kreitman skew (MKS) tests for an enrichment of non-synonymous mutations in either fixed differences indicative of directional (positive) selection (MKS < −1) or polymorphisms, indicative of balancing selection (MKS > 1) as measured by a 2 × 2 contingency table (see Methods). The genes were filtered to retain sequences where the absolute numbers of polymorphic or fixed differences were ≥5 and where the mean depth did not exceed 1.75 times the mean depth across all genes, so as to exclude possible duplicates (*n* = 4174). Under Fisher’s exact test, 271 of the filtered genes had a *p* value < 0.05. Of these sequences, 31 showed evidence of directional selection and 237 genes showed evidence of balancing selection (Supplementary Table S2). Gene ontology analysis among the 31 genes under directional selection showed that no terms were significantly over-representationed among these genes when a Bonferroni correction was applied; similarly no pfam domains were significantly enriched among these genes. Of the 237 genes showing evidence of balancing selection, 3 GO terms were significantly over-represented; cell junction protein (fold enrichment >5, *p* = 1.13 × 10^−2^), cell adhesion molecule (fold enrichment = 4.51, *p* = 1.59 × 10^−3^) and G-protein modulator (fold enrichment = 3.69, *p* = 1.24 × 10^−2^). Pfam enrichment of the positive MKT genes revealed immunoglobulin I-set domains to be enriched (PF07679, OR = 3, *p* = 0.02) and four dynein protein domains (PF03028, OR = 9, *p* = 0.009; PF08393, OR = 8, *p* = 0.01; PF12777, OR = 9, *p* = 0.009; PF12780, OR = 9, *p* = 0.009). When the HKAr and MKS distributions are overlapped, four genes are shown to have extreme scores in both tests (Fig. 3), included in these are heat shock 40 protein (*hsp40*), a homeobox protein, Elongator Complex Protein and an uncharacterised protein (full results shown in Supplementary Table S2).

### Historical demography of *S. mansoni*

Based on evidence from our phylogenetic analysis and PCA we constructed a putative population history for our samples using a number of models (Supplementary Fig. S2). We included African *S. mansoni* and *S. rodhaini* as two populations to estimate the divergence date between species, and effective population size (*N*_*e*_) before and after speciation. These posterior values are estimated through coalescent models as functions of two parameters; mutation rate and generation time. Our estimates of these parameters are discussed in the Methods and the Discussion.

Analysis of this model using G-PhoCS shows that the split between African *S. mansoni* sequences and *S. rodhaini* occurred 126.5 thousand years ago (KYA) (95% CI 107.5–147.6KYA) when migration is permitted from *S. mansoni* into *S. rodhaini* populations. This introgression is unidirectional and appears to have taken place soon after the initial divergence between *S. mansoni* and *S. rodhaini,* though at a low frequency (proportion of *S. rodhaini* population arising via migration from *S. mansoni* per generation = 5.53 × 10^−7^). Ancestral *N*_*e*_ before speciation (*N*_*e*_ = 4.25 × 10^5^) is shown to be more than four times greater than present *N*_*e*_ values for the extant African *S. mansoni* population as a whole (*N*_*e*_ = 1.02 × 10^5^) and more than 10 times greater than the *N*_*e*_ of extant *S. rodhaini* (*N*_*e*_ = 1.50 × 10^4^). No migration can be seen between *S. rodhaini* and any extant populations of *S. mansoni* in our models (Supplementary Fig. S2), which contrasts with recent evidence of hybridisation between natural populations of the two species in East Africa^36^,^37^, and suggests that these events have not occurred at a high enough frequency over the past 126.5KYA to be detected by our models.

We modeled the split between New World (Guadeloupe) and West African (Senegal and Cameroon) samples (Supplementary Fig. S2). We excluded the NMRI sample from this analysis as it has been kept as a laboratory isolate for hundreds of generations since it was isolated in the early 1940s^38^ and so violates the assumptions of the G-PhoCS model. The model gives a best estimate of the date of separation as 543 years ago; given that the Guadeloupe samples were isolated from the field in 1983 this is dated to 1440 AD (95% CI 1118–1743 AD). The historical date of the arrival of slaves to Guadeloupe, 1669 AD^39^, is therefore included within by the 95% confidence interval. Different permutations of this model (for instance modeling the split between New World and only the Senegal strain and the inclusion of migration bands) produced near-identical results.

Additional models were used to calculate the divergence between East (Lake Victoria and Lake Albert) and West (Senegal and Cameroon) African samples into different populations. On the basis of the phylogenetic tree (Fig. 2) and G-PhoCS evidence, we model the process with the Ugandan isolates as the basal clade, with Cameroon and Senegal splitting off (Supplementary Fig. 2). We date the split between Uganda and Cameroon to 5.92KYA (95% CI 4.71–7.16KYA). The *N*_*e*_ of the ancestral population shows no significant difference with Uganda (*N*_*e*_ = 1.11 × 10^5^ for both populations), whereas the Cameroon *N*_*e*_ showed a fourfold reduction since the split (*N*_*e*_ = 2.44 × 10^4^). Senegal split at a later date from the Ugandan isolates, 1.52KYA (95% CI 0.439–2.59KYA) and the effective population declines nearly tenfold (*N*_*e*_ = 1.44 × 10^3^). In both scenarios there is no evidence of migration in either direction following the population split. The *N*_*e*_ estimates of the G-PhoCS models are summarised in Table 2.

**Figure 4:**
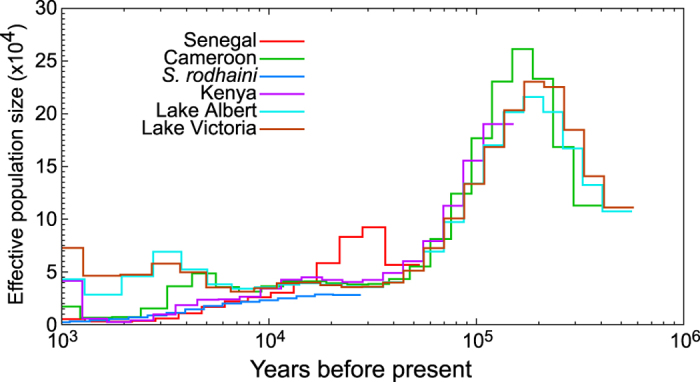
PSMC estimates of effective population size variation through time. PSMC estimates of *N*_*e*_ are shown for all African *S. mansoni* isolates for dates between 1,000,000–1,000 years before present. Bootstrap confidence intervals for these lines are show in Supplementary Fig. S3.

Effective population size estimates from this coalescent model assume a steady-state population existing along each branch of the population tree, and so cannot capture fine-grained demographic changes. Much finer temporal resolution of *N*_*e*_ is obtained by the PSMC model, in which each sample gives an independent estimate of how *N*_*e*_ has varied between discrete time periods. Results of this model largely corroborate those from G-PhoCS, with a large population size before 120KYA, when we infer that our *S. mansoni* and *S. rodhaini* alleles were present in the same ancestral population (Fig. 4, bootstrapping shown in Supplementary Fig. S3). The additional resolution of PSMC output reveals that a fall in *N*_*e*_ is then experienced by all sequences between 20–100KYA suggestive of a population bottleneck. A limitation of PSMC is that it estimates constant *N*_*e*_ within a time window, and so cannot differentiate between sudden and gradual reductions in population size and so the exact timing and rate of change in *N*_*e*_ is not possible to estimate exactly. In the last 7KYA *N*_*e*_ continues to decline in West African and New World populations, though *N*_*e*_ increases in the Lake Victoria and Lake Albert populations.

The PSMC results also corroborate some of the dates estimated by other methods. The demographic history of all isolates in the PSMC model follow the same trajectory until 7KYA ago, suggesting that *S. mansoni* existed as a single population until this point, approximately matching our estimate for the split between Uganda and Cameroon populations in G-PhoCS (95% CI 4.71–7.16KYA). The only deviation from this pattern is seen in the Senegal sample, which shows an increase in *N*_*e*_ 20–40KYA not present in the other isolates, though the cause of this difference is not clear. We also note that PSMC cannot help understand contemporary changes in population size. While our PSMC analysis has good resolution from 2–300KYA for the majority of sequences, more recently than 2KYA uncertainty increases as the number of coalescent events decreases, this is shown by the wide bootstrap values around the central tendency (Supplementary Fig. S3).

## Discussion

We estimate the split between the New World (Guadeloupe) and West African populations (Senegal and Cameroon) to be between 1117–1742 AD. The two proposed possible timings of the Africa –New World split are when the continents split 80–120 million years ago or during the Slave Trade of the 16–19^th^ Centuries. The confidence interval we have obtained spans the historical date of the earliest slaves to the New World. Slaves from French Colonies in West Africa were exported to islands in the French Caribbean, including Guadeloupe, from 1669 until 1864 and the total number of Africans taken from West Africa by French ships to Guadeloupe during this period numbered over 22,000^39^. Our result shows the data from whole genome sequences to be consistent with both historical dating and the results found from previous studies on *S. mansoni* using fewer loci.

Genomic and archaeological evidence from other pathogens suggests that many human infectious diseases originated in the Old World and migrated to the New World^40^, though the role of humans in transmitting diseases between continents is contested. For instance *Mycobacterium tuberculosis,* which originated in Africa, was found in Peruvian remains dated to Pre-Columbian times. Based on genomic data, seals were implicated as the most likely reservoir that transmitted *M. tuberculosis* from Old to New World^41^. Diseases such as syphilis, caused by the spirochete *Treponema pallidum*, are assumed to have been transmitted to the New World with the Conquistadors of the 16^th^ Century and this is supported by archaeological findings^42^, though molecular data is lacking. The protozoan parasite *Leishmania chagasi*, which causes visceral leishmaniasis in the New World, is also thought to have been brought during the time of the Conquistadores though dogs imported into the Americas ~500 years ago, a migration event which caused a genetic bottleneck that has been observed in microsatellite loci^43^. The evidence presented here builds on the theory that many human infectious diseases originated in the Old World and future population genomic studies of other pathogens have a valuable role to play in understanding the historical patterns of transmission that have shaped the current distributions of infectious diseases.

The evidence from coalescent models suggests that the emergence of *S. mansoni* as a distinct species occurred around 126.5KYA. This estimate is an order of magnitude earlier than previous estimates that placed the split at 2.8MYA and 1.2–10MYA respectively^20^,^21^. We propose that this most likely occurred in East Africa as the Ugandan isolates are the earliest diverging *S. mansoni* samples on the maximum likelihood tree. This supports the conclusions drawn from previous mtDNA barcoding studies^21^. This is also reflected in the fact that our sequences from Lake Victoria and Lake Albert show the highest number of variants, indicating they have had the longest separation from the New World reference genome. *A priori* we might expect the origin of *S. mansoni* to be in East Africa as *S. rodhaini*, which shares a common ancestor with *S. mansoni*, has its present range in this part of the world. Furthermore, East Africa is where the earliest paleontological evidence for anatomically modern humans is found. A series of subsequent paleontological sites in this region indicate that early human populations inhabited this region throughout the late Pleistocene^44^,^45^.

**Figure 5:**
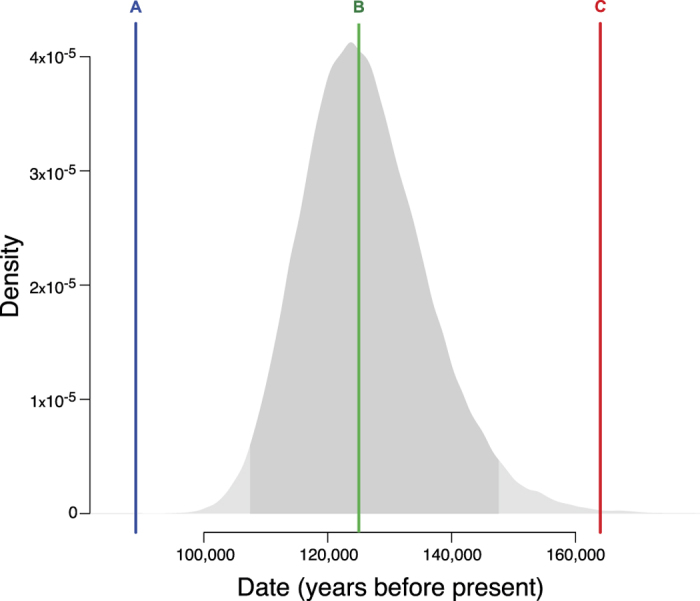
Date of *S. mansoni* – *S. rodhaini* divergence and archaeological evidence for fishing in Africa. The posterior probability distribution for the date of the last common ancestor of *S. mansoni* and *S. rodhaini* from G-PHoCS (in grey), with the 95% highest posterior density confidence interval in darker shading. Coloured lines show central estimates for the dates of three early fishing sites in Africa, at (**A**) Upper Semliki Valley, Democratic Republic of Congo (dated to between 74 and 111KYA)^50^, (**B**) Red Sea Basin, Eritrea (dated to between 118 and 132KYA)^47^, (**C**) Mossel Bay, South Africa (dated to between 152–176KYA)^49^.

We hypothesise that the adoption of fishing by human communities may have triggered the speciation of *S. mansoni* and *S. rodhaini* as this led to hunter-gatherer groups residing for longer periods by water bodies, leading to the establishment of *S. mansoni* transmission. Archaeological evidence from across Africa indicates a gradual accumulation in technology during the Middle Stone Age with the earliest evidence for exploiting aquatic resources from sites in South Africa, Eritrea and the Democratic Republic of Congo (DRC)^46^,^47^,^48^,^49^. The earliest evidence is from Mossel Bay, South Africa where stone artefacts and substantial shellfish deposits are dated to 152–176KYA^49^. Similarly, at the Red Sea Basin in Eritrea deposits of stone tools and shells from marine organisms have been dated to 118–132KYA^47^. Early exploitation of freshwater resources is also seen in the Upper Semliki Valley, DRC where bone assemblages suggest that a well developed industry to process fish in Central Africa was established 74–111KYA^48^,^50^. Our posterior distribution for the speciation of the last common ancestor of *S. mansoni* and *S. rodhaini* overlaps with these archaeological dates (Fig. 5), suggesting that changes to subsistence patterns in Africa around this time may have led to increased water contact and subsequently human infection with schistosomes.

The PSMC models show that the ancestors of all extant *S. mansoni* populations underwent a bottleneck between 20–100KYA. These changes may be driven by changes to human populations, the effect of climate on the freshwater habitats of the intermediate snail host, or a combination. Coalescence modeling of human populations in Africa support a decline in *N*_*e*_ from 50–100KYA, though this bottleneck is milder than in Non-Africans^51^. The East African climate was generally arid from 60KYA until the beginning of the Holocene^52^ and fluctuations in temperature and precipitation throughout this period impacted on the size and depth of East African Lakes, where we presume the ancestral population of *S. mansoni* existed prior to 7KYA. Geological evidence suggests that Lake Tanganyika went through three phases of low lake levels at the end of the Pleistocene (35–40KYA, 23KYA and 18KYA) and Lake Victoria was almost completely dry between 12–17KYA. The lake levels all began to rise rapidly about 11KYA, reaching levels close to those observed in the present^53^. As PSMC is unable to specify exactly when in the 20–100KYA window the bottleneck in *S. mansoni* occurred, we are not able to associate the fall in parasite *N*_*e*_ with any specific event. Therefore, climatic fluctuations, a fall in human *N*_*e*_ and changes to lake levels are all plausible explanations for the reduction in *S. mansoni* population size at the end of the Pleistocene.

**Figure 6:**
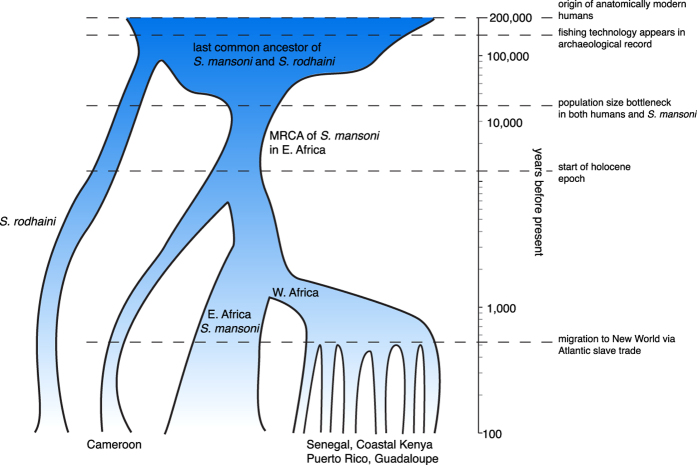
Summary of *Schistosoma mansoni* population history. Widths of branches represent approximate log *N*_*e*_ (effective population size. Note timescale is drawn on a log axis.

By combining evidence from our phylogenetic tree with coalescence modeling we argue that *S. mansoni* originated in East Africa and that the ancestors of all extant populations remained there as a single population until 7KYA as PSMC output shows all populations follow the same demographic trajectory from 7–120KYA. More recently than this, the populations follow different paths as *N*_*e*_ rises in Lake Albert and Lake Victoria isolates and falls for New World and West African isolates. We hypothesise, therefore, this was around the time that populations of *S. mansoni* began to migrate across the continent and colonise areas of West Africa. This view is supported by evidence from G-PhoCS, which shows that Cameroon split away from the East African population 6KYA years ago, followed by the Senegal population 1.5KYA. Movement of human populations in Africa may explain the migration of parasites to new regions. The Bantu expansion was the most significant movement of peoples around Africa following the start of the Holocene followed by the Yoruba and Luhya populations expanding from 6KYA out of West Africa into Central, Southern and Eastern Regions^54^. Both the increase in human population size and the extensive movement of peoples across Africa following the milder conditions of the Holocene may have created conditions in which schistosome parasites could be spread through water sources as humans migrated across the continent. The population history of *S. mansoni* based on our models is summarised in Fig. 6.

Both coalescence models presented here use two parameters, generation time and mutation rate. We have made estimates of both these values as 0.2 years (see Methods) and 8.1 × 10^−9^ per basepair per generation (Supplementary Fig. S4) respectively^55^,^56^,^57^. Generation time, which includes the latency period in humans, the latency period in snails and the average time to transmit between the two hosts, varies with water temperature of the snail intermediate host, the species of snail and the force of infection^58^. Given the variability of such factors spatially and temporally, our estimate of the generation time is a simplification. Our estimate of the mutation rate, based on the relationship between genome size and mutation rate, may be subject to alteration on the basis of experimental evidence^59^. Therefore if revisions are made to either the mutation rate or generation time in the future, both dates of divergence and *N*_*e*_ estimates provided here will need revision. Despite these limitations, we find it encouraging that our estimate of the timing of migration to the New World overlaps with historical dating, suggesting that our estimates of the parameters are likely to be robust.

Our sample from coastal Kenya shows a pattern in the phylogenetic tree and PCA significantly different to that expected given its distant geographical position from the Puerto Rican reference and proximity to Uganda. In all instances the Coastal Kenya isolate clusters with the New World and Senegal strains, as opposed to the other East African sequences. Previous phylogenetic analysis has shown that Coastal Kenya and Zambia form a distinct clade away from other East African parasites on the basis of a 451bp region of the cox1 gene in the mitochondria^9^. This suggests that Coastal Kenya and Zambian *S. mansoni* have had a separate evolutionary history to parasites in the African great lakes, which may be due to separate histories of these regions. Throughout the 19^th^ Century slaves were traded between Portuguese colonists along the Zambezi and Arab traders in the Horn of Africa predominantly via the port of Mombasa^60^.Therefore a movement of people, and presumably parasites, occurred between these two regions, which may have contributed to their differentiation with other *S. mansoni* samples within East Africa. Denser sampling of the diversity of parasites in both East and West Africa will be needed to resolve the history of these parasites.

Through population genetic tests where we specify *S. rodhaini* as the out-group, we have highlighted genes that show a deviation from neutral expectations, either through an enrichment of non-synonymous substitutions or an over-representation of polymorphisms relative to fixed differences. Using a branch model of *d*_*N*_*/d*_*S*_, we found 767 genes showing evidence of positive selection (*d*_*N*_*/d*_*S*_ > 2); of these a significantly enriched category of genes, ECM proteins, were found to have an increased level of expression during a period of host interaction in the schistosome lifecycle (24 hour schistosomula stage). This suggests that these genes may contribute to host invasion, as they have undergone rapid evolution as the parasite *S. mansoni* has become human-infective. When we tested for significance under a likelihood ratio framework, we found VAL21 and a cercarial elastase gene to be under significant positive selection. The VAL genes excrete protein products that induce allergic responses. Transcriptomic evidence has shown that VAL21 is expressed primarily in the cercarial stage, suggesting it may play a role in invasion of the definitive mammalian host^32^. Cercarial elastase cleaves insoluble elastin, a major component of the dermis, and is the only protease required for mammalian host invasion^33^. While cercarial elastase genes are broadly conserved within the genus *Schistosoma,* our finding of a signal of positive selection in Smp_119130 may hold the key for human-specific invasion.

Gene with high values in the MKS, suggestive of balancing selection, show enrichment for pfam domains found in immunoglobulin I-set proteins, a domain linked to cell-cell interactions that could facilitate adaptation to the host. Dynein-domain containing proteins were also significantly enriched; this motor protein is involved in transport of organelles in cells, suggesting that the energetic requirement of *S. mansoni* cells is a target for selection^61^. As the isolates are not from first passage (see Methods), this represents a potential limitation on our analysis for selection if passages have introduced novel selection pressures. This highlights the importance of using whole-genome sequences directly from clinical isolates in future studies.

Combining the MKS and HKA test revealed genes with extreme values, indicative of balancing selection. One of the main outliers is hsp40, part of a family of proteins known to regulate hsp70s, a gene family that encodes proteins that protect cells from stress and that have undergone an expansion in other platyhelminth species, such as tapeworms^62^. While there may be multiple explanations for the pattern of variation observed on any particular gene, the loci we identify are subject to unusual evolutionary processes compared to the rest of the genome and so may provide valuable targets for future functional experiments. As with most other non-model organisms, inferring the function of ‘hits’ from our tests for selection can be difficult. Efforts are therefore underway to develop high-throughput functional genomic approaches to flatworm biology^63^ and the genes that we have identified as outliers in the selection tests from this analysis will be fed into an ongoing RNAi screen to examine function.

## Methods and Materials

### Samples

Samples (Table 1), with the exception of NMRI, came from the schistosomiasis collection (SCAN) at the Natural History Museum (NHM), London^64^. All schistosomes provided by SCAN were collected by NHM staff in collaboration with in-country partners, except for the Guadeloupe specimens, which were originally provided by Dr André Thèron, Université de Perpignan, France. SCAN isolates were collected from field sites within infected *Biomphalaria* snails, which were shed in the laboratory and the cercariae used to infect mice. SCAN and Guadeloupe isolates were passaged between 2 and 15 times. The NMRI sample is a Puerto Rican isolate from the early 1940s that has subsequently been maintained in the laboratory for hundreds of generations^38^ and was used to build the reference genome for *S. mansoni.* Adult worms were extracted from mice and kept in liquid nitrogen storage prior to DNA extraction. For each isolate a single adult worm was used for DNA extraction and sequencing.

### Sequencing

The libraries were sequenced on the Illumina Genome Analyser IIX for 76 cycles in each direction (paired-end reads) using V4 SBS sequencing kit and proprietary reagents according to manufacturer’s recommended protocol (https://icom.illumina.com/). ENA Accession numbers for each sample are shown in Table 1.

### Variant Calling Pipeline

Reads were mapped against v5.2 of the *S. mansoni* reference genome^30^ using smalt (http://www.sanger.ac.uk/resources/software/smalt/) using options (-a -o “-i 2000 -r 1 -x -y 0.85” -k 13 -s 2). This resulted in reads mapping to the region of highest similarity in the reference genome above an 85% similarity threshold. When reads could be mapped to multiple locations with equal similarity one position was randomly chosen, though these repetitively mapped reads are excluded by our variant calling. Processing of the resulting bam files, prior to variant calling, was performed using GATK version 2.6 to remove duplicate reads and perform local realignment around indels^65^. Single nucleotide polymorphisms (SNPs) were called using three algorithms; HaplotypeCaller and UnifiedGenotyper (implemented in GATK) and samtools version 0.1.19 mpileup^66^. Filtering was applied to each set of variants; SNPs with genotype quality (GQ) scores (a measure of the confidence of the SNP call) <10 and read depth (DP) <10 were removed. Lastly, the intersection of the three calling algorithms was found using bedtools version 2.17.0.

### *d*_*N*_ */d*_*S*_ ratio

The 11727 coding sequences in the *S. mansoni* genome were filtered by removing alternative transcripts (1005 sequences removed), genes with low coverage in the *S. rodhaini* alignment (<75% of bases covered at 10×, 719 sequences removed), genes with excessive coverage (>1.75 × mean depth, 475 sequences removed) and gene models with an incorrect number of nucleotides (not divisible by three, 14 sequences removed) leaving a filtered subset of 9514 coding sequences for downstream analysis. Maximum-likelihood estimates of *d*_*N*_*/d*_*S*_ for each protein-coding gene were calculated by running codeml from the PAML package^67^ in branch mode, where the phylogenetic tree for African *S. mansoni* and *S. rodhaini* was specified and partitioned into two clades, separating the branch leading to the S. rodhaini sequence (the ‘foreground’ branch) from all other branches between different *S. mansoni* samples. We ran two scenarios for each coding sequence, in which the value for *d*_*N*_*/d*_*S*_ on the foreground branch was fixed at 1 (null hypothesis) or unfixed (alternative hypothesis) and the log likelihood (lnL) in both scenarios compared using the likelihood ratio test. We calculate the test statistic *D*, where *D* = −2*(lnL_fix_ -lnL_unfixed_). The test statistic is then compared against a Chi-Square distribution, where degrees of freedom *(df)* are equal to the difference in the number of parameters in the two models *df* = params_unfixed_-params_fixed_, for all coding sequences in our dataset *df* = 1. We set the significance threshold for the Chi-Square distribution at *p* < 0.05. Statistical overrepresentation analysis was conducted using PantherDB (version 10.0)^68^.

### Hudson-Kreitman-Aguade (HKA) Ratio

The HKA ratio is the difference in the ratio of polymorphic (*π*) and fixed differences (*K*) within a gene. Where *π*/*K* is high, therefore, this indicates balancing selection (ie diversity within African *S. mansoni*) and when low and enrichment of interspecific nucleotide divergence between African *S. mansoni* and *S. rodhaini*. The HKA test was implemented through the use of previously developed software^69^.

### McDonald-Kreitman Test

Polymorphic differences within populations (African *S. mansoni*) and fixed substitutions between populations (African *S. mansoni* vs *S. rodhaini*) were identified using previously developed software^70^. The MK skew, or neutrality index, is defined as log_2_ ((*Ns*_poly_/*S*_poly_)/(*Ns*_fix_/*S*_fix_)), where *Ns* and *S* are the number of non-synonymous and synonymous sites within a coding sequence, and subscripts *fix* and *poly* refer to fixed differences between populations and polymorphisms within a population respectively. To avoid divide by zero errors, 1 was added to all scores. GO term enrichment was conducted in PantherDB^68^ and enrichment of pfam domains was performed using a one sided Fisher’s exact test in R (version 3.2.2).

### Consensus fasta files

The phylogenetics software package RaxML and coalescence models PSMC and G-PhoCS require fasta sequences, so in the absence of phase information for our samples we calculated a consensus sequence of the diploid genotypes. These were generated from the filtered vcf files using a modified perl script. Heterozygotes are represented by IUPAC ambiguity codes. These files were filtered to remove mitochondrial sequences, scaffolds not aligned to chromosomes and the scaffold representing shared sequence between the Z and W chromosomes^30^: this left the scaffolds aligned to the 7 autosomal chromosomes. To ensure loci included in G-PhoCS were unlinked and sufficiently small that little recombination occurs within loci, we examined the decay in linkage disequilibrium (LD) with distance between pairs of SNPs in our data using SNP data in vcf files, which were phased using SHAPEIT2 (v 2.790)^40^, and analysed using VCFtools (v0.1.11) –hap-r2 and found that LD decays to background levels for distances great than around 400kb (Supplementary Fig. S5). To be conservative, we thus divided our consensus calls into 1kb blocks separated by 500kb. These same input regions were concatenated and used as input for RAxML.

### Phylogenetic Tree

We constructed a Maximum likelihood (ML) tree using RAxML version 8.0.24^71^ under a GTR model of nucleotide substitution with a gamma distribution of rate variation across sites^71^. For each run, twenty randomly generated starting trees were used, and support for partitions are estimated as proportions of 100 bootstrap replicates displaying the partition. The final ML tree with bootstrap values was visualised in FigTree (v1.4.0)^72^.

### Principal Component Analysis

We built a matrix where every variable site in the genome is classified as homozygous reference (0), homozygous alternative (1) or heterozygote (0.5). This matrix was transposed in R version 3.1.3 and the PCA was performed with the prcomp function with all default settings retained.

### Estimates of parameters for coalescent models

Mutation rate was calculated through a linear regression of the relationship between log-genome size and log-mutation rate^73^. The best fitting model for mutation rate μ in bp was *mu* = 2.585324 × 10^−10^ × *g*^0.584^ for genome size *g* in Mb, giving an estimate of 8.1 × 10^−9^ per bp per generation (Fig. S4). Generation time estimated by adding the time taken for the parasite to start producing eggs after infection with human host (~42 days)^55^ with the latency period in snails (~28 days) +time for transition between the two hosts (1 day) = ~71 days (0.2 years). There is a wide variation around the generation time estimates, as latency period in snails is temperature and species dependent within the *Biomphalaria* genus. To obtain effective population sizes (*N*_*e*_) in numbers of individuals, we used the relationship *N*_*e*_* = θ*/(4μ) where *θ* is the uncalibrated population size and μ is the mutation rate. To obtain the divergence time in years (D), we used the relationship, D = (0.2τ)/μ where τ is the uncalibrated divergence time.

### Generalised phylogenetic coalescence sampler (G-PhoCS)

G-PhoCS is used to estimate several key population parameters; ancestral population size, divergence times and gene flow between species^28^. We ran a number of different G-PhoCS analyses to test how sensitive estimates of these parameters are to different assumptions about population structure and migration (Fig. S2). Each simulation was run for a minimum of 10 million iterations, with at least 20 parallel chains and until convergence was reached (effective sample size (ESS) for all parameters >200). Samples from the MCMC chains were also manually inspected in Tracer (v1.5) to ensure chains had reached convergence.

### PSMC

The Pairwise Sequentially Markovian Coalescent (PSMC) model uses the local density of heterozygous sites to infer the time to most recent common ancestor (TMRCA) for blocks of the genome, effective population sizes for discrete historical time windows and the positions of transitions between TMRCAs (i.e. boundaries of blocks) are also inferred from data. Modelling only a single diploid genome and using a computationally convenient approximation to the full coalescent makes treating the coalescent with recombination tractable for genome-scale data^27^. Because data from only a single diploid genome is used, the method loses accuracy more recently in time, eg for humans it is not informative about demographic events more recently than 20KYA. PSMC was used with data from each field isolate of *S. mansoni*, each time using parameters -N50 -t30 -r4 -p “26*2 + 4 + 6”, with the distribution of TMRCA segments chosen to ensure at least 10 recombination events occur in each segment, as suggested in the PSMC manual, on a preliminary set of variant calls from mpileup for these data. Bootstrapping was performed 100 times for each sample (Supplementary Fig. S3).

## Additional Information

**How to cite this article**: Crellen, T. *et al.* Whole genome resequencing of the human parasite *Schistosoma mansoni* reveals population history and effects of selection. *Sci. Rep.* **6**, 20954; doi: 10.1038/srep20954 (2016).

## Supplementary Material

Supplementary Information

Supplementary Table S1

Supplementary Table S2

## Figures and Tables

**Table 1:** Isolates sequenced in the study.

ID	Country	ENA Accession	Year	Reads	Median Coverage	MP	UG	HC	Overlap	Heterozygotes (%)
NMRI	Puerto Rico	ERS039722	1940	16684649039	43	547791	585813	532965	459938	265341 (57.7)
Senegal	Senegal	ERS074979	1998	22441782383	57	807680	843282	764845	661528	118616 (17.9)
Cameroon	Cameroon	ERS074981	1997	16506961647	42	1555732	1599021	1491934	1332792	879648 (66.0)
Coastal Kenya	Kenya	ERS074980	1996	22968467451	58	931195	971723	891959	793274	373547 (47.1)
Lake Albert	Uganda	ERS074982	2004	21755312204	55	2236138	2292720	2142399	1951022	1216415 (62.3)
Lake Victoria	Uganda	ERS141391	2005	20094431877	50	2384792	2438722	2281572	2043541	1241072 (60.7)
Guadeloupe 1	Guadeloupe	ERS427393	1983	8201011511	23	966028	987299	933593	772668	508996 (65.9)
Guadeloupe 2	Guadeloupe	ERS427392	1983	10900000000	30	983954	1012647	951671	830275	541482 (65.2)
Guadeloupe 3	Guadeloupe	ERS427394	1983	12618861609	35	1046797	1083754	1009148	872007	557070 (63.9)
Guadeloupe 4	Guadeloupe	ERS427395	1983	8560020250	24	961926	986457	927716	750295	470137 (62.7)
*S. rodhaini*	Burundi	ERS076740	2002	11508748607	28	11246613	11772768	10948674	8102216	404373 (5.0)

Table shows number of reads mapped to the *S. mansoni* reference genome v5.2, median coverage attained and SNPs called by mpileup (MP), UnifiedGenotyper (UG), HaplotypeCaller (HC) and the consensus result (Overlap). The numbers of heterozygous SNPs from the consensus results are shown. The year column indicates the date of first passage.

**Table 2:** Effective population size estimates obtained from G-PhoCS.

Population	*Mean N*_*e*_(× 10^4^)	95% CI (× 10^4^)
LCA *S. mansoni*/ *S. rodhaini*	42.8	38.7–46.7
Ancestral *S. mansoni*	10.2	9.77–10.7
Present *S. rodhaini*	1.47	1.26–170
Present East Africa (*S. mansoni)*	6.50	3.67–9.35
Present West Africa (*S. mansoni)*	0.700	0.330–1.09
Present Guadeloupe *(S. mansoni)*	0.307	0.147–0.473

Values shown are mean of the posterior probability distribution and upper and lower bounds of the 95% highest posterior density confidence intervals obtained by the model. Note that ‘present’ values are average of *N*_*e*_ along branches. All values are given to 3 significant figures.
