# Supplementary material for: Whole genome resequencing of the human parasite Schistosoma mansoni reveals population history and effects of selection
Source: Sci Rep. 2016 Feb 16;6:20954. doi: 10.1038/srep20954 (PMC4754680; doi:10.1038/srep20954)
Supplement: Supplementary Information [file srep20954-s1.pdf]

Whole genome resequencing of the human parasite *Schistosoma mansoni* reveals population history and effects of selection

Thomas Crellen[1,2,4], Fiona Allan[3], Sophia David[2], Caroline Durrant[2], Thomas Huckvale[2], Nancy Holroyd[2], Aidan M. Emery[3], David Rollinson[3], David M. Aanensen[1,2], Matthew Berriman[2], Joanne P. Webster[1,4], James A. Cotton[2]

1. Department of Infectious Disease Epidemiology, Imperial College London, St Mary's Campus, Norfolk Place, London W2 1PG, United Kingdom
2. Wellcome Trust Sanger Institute, Hinxton, CB10 1SA, United Kingdom
3. Department of Life Sciences, Natural History Museum, London, SW7 5BD, United Kingdom
4. Department of Pathology and Pathogen Biology, Royal Veterinary College, University of London, Hertfordshire, AL9 7TA, United Kingdom

Supplementary Table S1. (separate file). Results of d_N/d_S tests for selection between *S. mansoni* and *S. rodhaini.* Results show branch model output from codeml and the likelihood ratio test results for each gene where d_N/d_S is fixed at 1 (null hypothesis) or allowed to vary (alternative hypothesis).

Supplementary Table S2. (separate file) Results of selection for coding regions in African *S. mansoni* when compared against *S. rodhaini* outgroup. Columns 2-7 show output from MK test and 8-10 show output from HKA test.

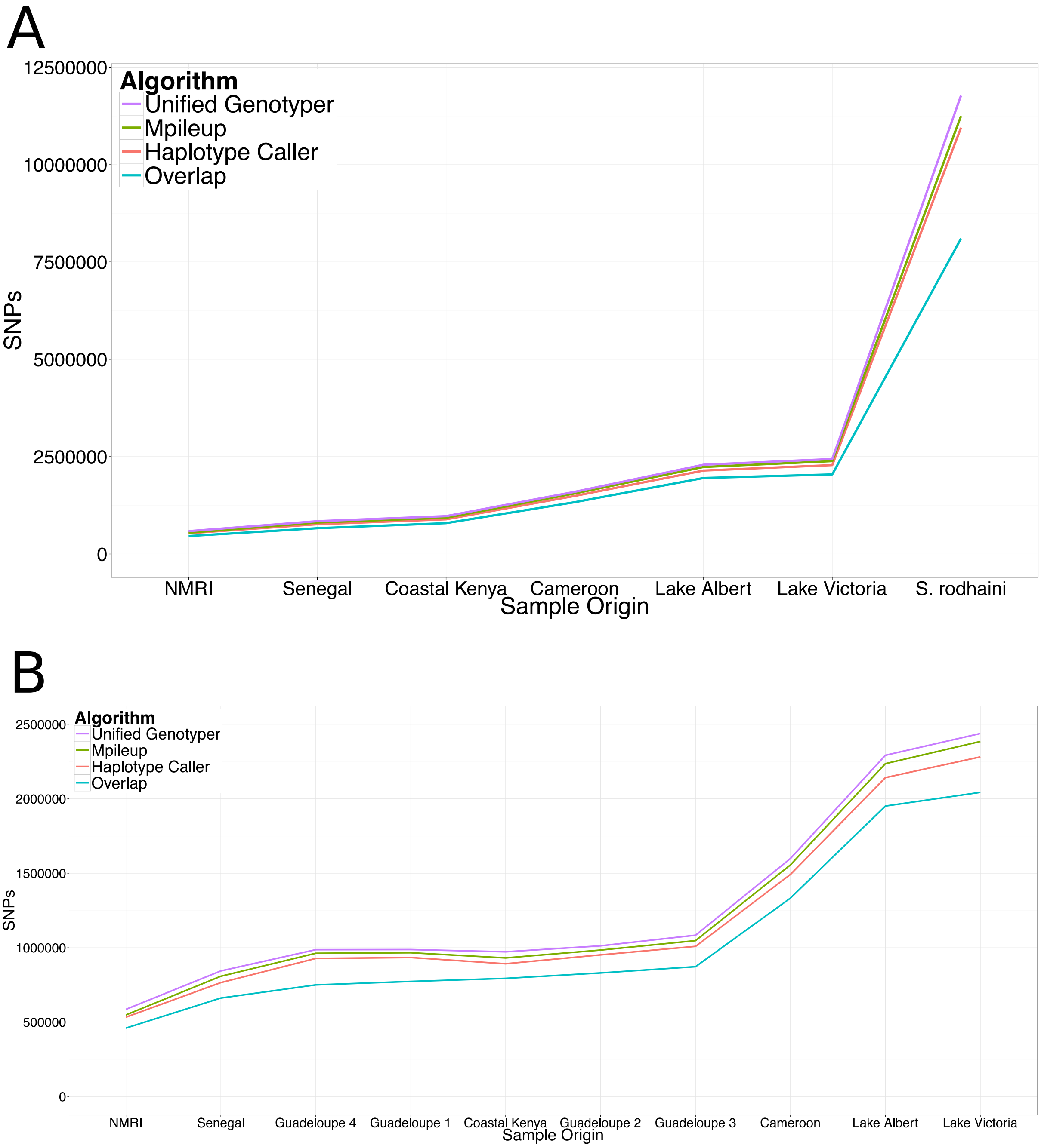

Supplementary Figure S1. Number of SNPs when samples called against *S. mansoni* reference V5.2[30] by calling algorithm. Consensus result (overlap) obtained by taking only those SNP sites called by all 3 callers A) shows results for all samples (*S. mansoni* & *S. rodhaini*) B) shows results only for *S. mansoni* samples

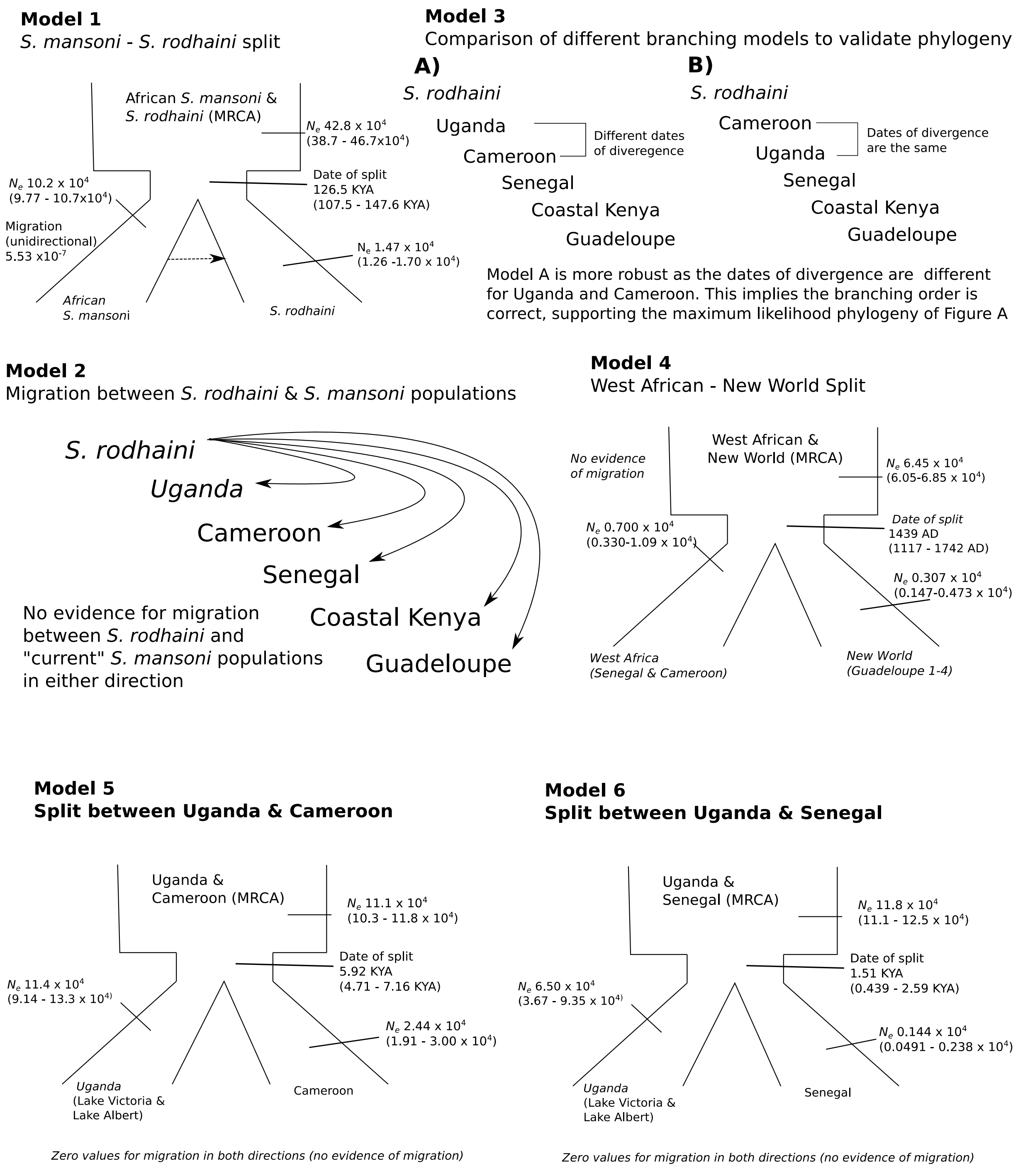

Supplementary Figure S2. Models for G-PhoCS coalescence simulations (1-6). Parameters set as: Migration Rate Alpha = 0.02, Migration Rate Beta = 0.0001, Tau Initial = 0.005, Tau Beta = 20000

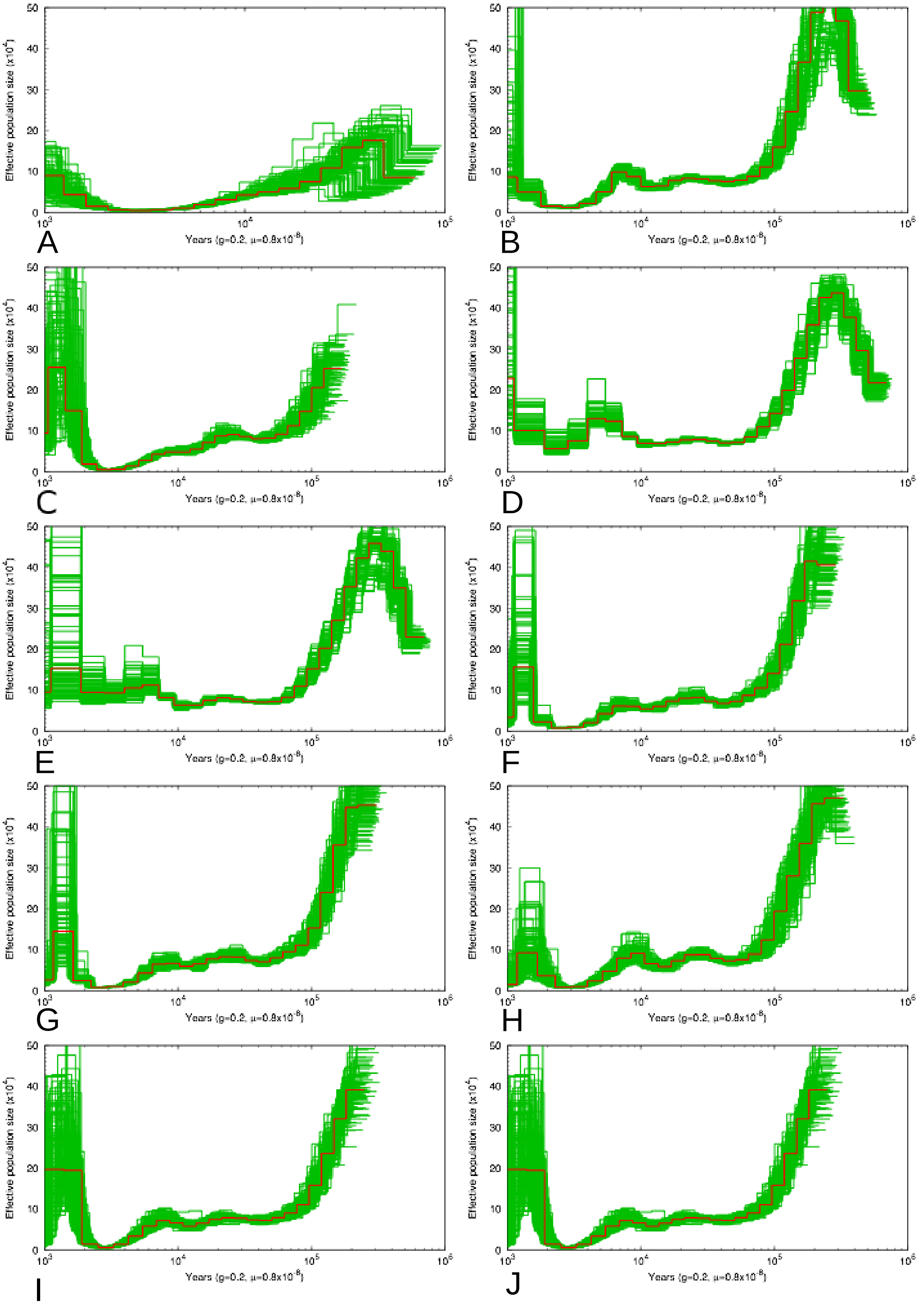

Supplementary Figure S3. Bootstrap confidence intervals (100 replicates) around PSMC estimates

A) Senegal B)Cameroon C) Coastal Kenya D) Lake Albert E) Lake Victoria F) Guadeloupe 1 G) Guadeloupe 2 H) Guadeloupe 3 I) Guadeloupe 4 J) *Schistosoma rodhaini*

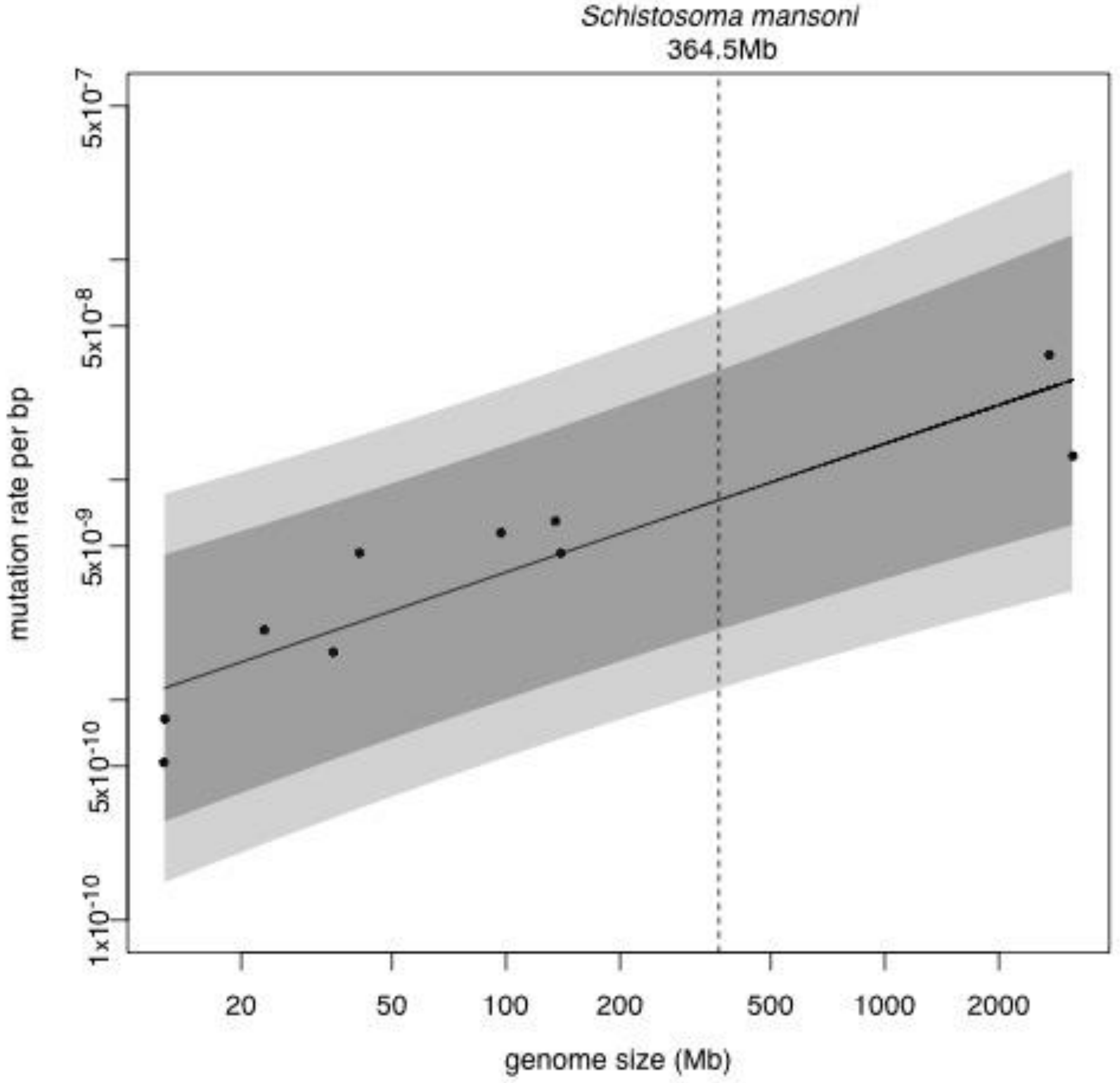

Supplementary Figure S4. Estimation of the mutation rate (per basepair per generation) of *S. mansoni* (dashed line) from genome size in Megabases as linear regression based on known values for other organisms[73]; 95% and 99% confidence intervals are shown as dark and light grey bands respectively.

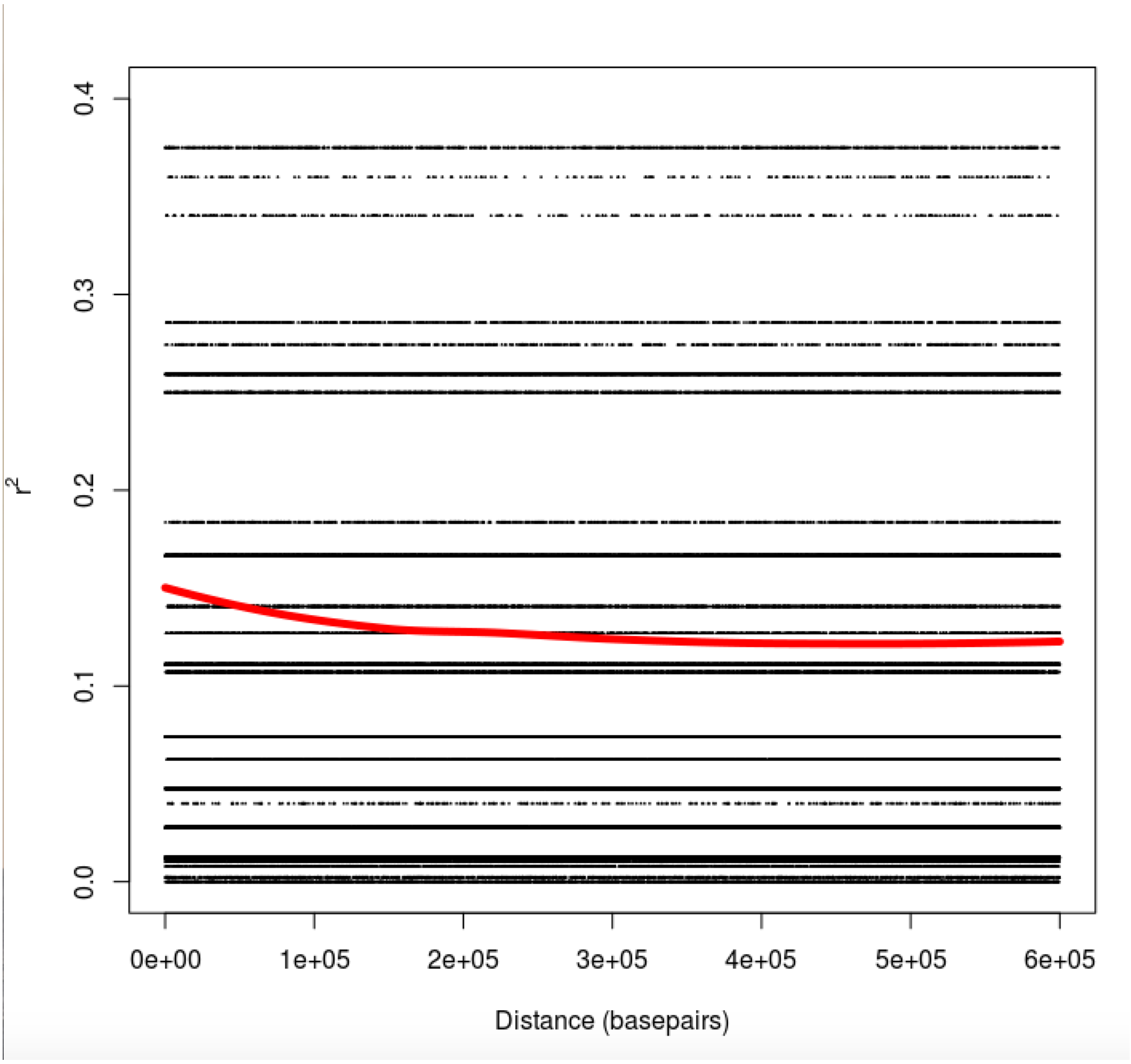

Supplementary Figure S5. A measure of linkage disequilibrium (r^2) across the *S. mansoni* genome, calculated from phased SNP calls. A loess line (red) is plotted to the data. The r^2 remains constant after a distance of approximately 400,000 base pairs, thus loci more than this distance apart are assumed to be in linkage equilibrium and evolve neutrally.
